# The influence of eye model parameter variations on simulated eye-tracking outcomes

**DOI:** 10.16910/jemr.16.3.1

**Published:** 2023-10-16

**Authors:** Joshua Fischer, Johan van der Merwe, David Vandenheever

**Affiliations:** Stellenbosch University, South Africa; Mississippi State University, USA

**Keywords:** Eye movement, eye-tracking, simulation, gaze, individual differences, synthetic images

## Abstract

The simulated data used in eye-tracking-related research has been largely generated using normative eye models with little consideration of how the variations in eye biometry found in the population may influence eye-tracking outcomes. This study investigated the influence that variations in eye model parameters have on the ability of simulated data to predict real-world eye-tracking outcomes. The real-world experiments performed by two pertinent comparative studies were replicated in a simulated environment using a highcomplexity stochastic eye model that includes anatomically accurate distributions of eye biometry parameters. The outcomes showed that variations in anterior corneal asphericity significantly influence simulated eye-tracking outcomes of both interpolation and model-based gaze estimation algorithms. Other, more commonly varied parameters such as the corneal radius of curvature and foveal offset angle had little influence on simulated outcomes.

## Introduction

The performance of an eye-tracker can be affected by a multitude of factors of which the variation in interpersonal eye biometry found in the population is thought to be a major contributor (16; 7). This makes developing an eye-tracker that performs well on a significantly large portion of the population a difficult endeavour.

Interpersonal variations in the performance of eye-tracking methods have been widely reported. Lai et al. (20) reported interpersonal variations in gaze estimation errors as large as 0.7°. Villanueva and Cabeza (33) reported errors larger than 2° between participants for a model-based gaze estimation algorithm using a similar eye-tracking hardware configuration. A series of studies investigating interpolation-based gaze estimation methods also reported significant interpersonal variations in eye-tracking performance (5; 6; 7).

Simulated eye-tracking data are generated by replicating the hardware and user of a device in a simulated environment. Ray-tracing operations are then used to simulate the position of features used by an eye-tracker such as the pupil and glints of the user on the image sensor of a camera model. Contemporary simulation environments also include realistic head models which allow the simulation of synthetic images of the entire eye region (32; 19, 22).

Using simulations, large and diverse eye-tracking datasets can be generated in a fraction of the time it would take to generate an equivalent amount of data in user studies (32). These datasets facilitate the rapid, repeated, and independent evaluation of eye-tracking methods. This is particularly advantageous during the early development of an eye-tracker in which various configurations of hardware and algorithmic components are considered (33; 23).

Increasing the interpersonal variations in simulated eye-tracking data has been one of the central ambitions in the development of increasingly complex synthetic image data. This is evidenced by the increasing number of realistic eye-region textures and complexities of the methods used to simulate variations in the structure of the eye-region from the initial development of synthetic eye-tracking images by Świrski and Dodgson (30) to more contemporary works such as the models developed by Wood et al. (32) and Stengel et al. (2019). However, a potential source of eye-tracking errors as fundamental as variations in eye biometry have been largely overlooked in pursuit of increasing the interpersonal diversity in simulated eye-tracking data.

Investigations that endeavour to include variations in eye biometry in simulated data have a few options. The simplest method is the one-at-a-time approach in which one parameter of an eye model is systematically varied within reported biometric ranges while keeping the other parameters constant. This method allows researchers to independently investigate the influence of each eye parameter on simulated outcomes (31; 15). Another method is to simultaneously generate a combination of eye model parameters from anatomically observed distributions (17) These approaches should be used with caution as they are likely to produce combinations of ocular biometry that are not biometrically plausible (29). The realism of the data is also limited by the complexity of the eye model of which the parameters were varied. For example, varying the parameters of an eye model that includes a single spherical cornea surface, as most studies in the eye-tracking-related literature have done, does not include realistic variations in the asphericity of the cornea in the simulated data.

Stochastic eye models are a promising alternative that can be used to include variations in eye biometry in simulated eye-tracking data using a high-complexity eye model. A stochastic eye model is developed by measuring several anatomical parameters of the eyes of a large population. The measured biometry is then used to develop a statistical model that can generate an infinite number of random biometrically plausible eye models with a distribution of parameters indistinguishable from the population from which the model was derived. Despite the availability of stochastic eye models such as those proposed by Rozema et al. (28) and Rozema et al. (29), stochastic models have not been considered in eye-tracking-related literature.

The combination of reported interpersonal variation in eye-tracking outcomes, the prevalence of simulated eye-tracking data, and the lack of eye biometry diversity included in the simulated data used throughout the eye-tracking-related literature motivate the need for an investigation of the influence that variations in eye model parameters have on the predictive power of simulated eye-tracking data. The combination of these findings suggests that the development of eye-tracking methods informed by simulated eye-tracking data may be hamstrung by a lack of eye biometry distributions in the data. This may have contributed to the interpersonal variations in eye-tracking performance observed in real-world eye-tracking outcomes.

This study investigated the influence that variations in eye model parameters have on the ability of simulated data to predict real-world eye-tracking outcomes. A stochastic eye model was used to generate unique eye models with biometrically plausible distributions of eye model parameters. The real-world experiments performed by Guestrin and Eizenman (14), and Blignaut (6) were then simulated using each eye model. These two comparative studies were chosen so that the influence of interpersonal variations in eye model parameters on both interpolation and model-based gaze estimation algorithms could be investigated. The main findings of each comparative study were identified, and the simulated outcomes were compared to the real-world experimental outcomes of the comparative studies. A multivariate regression analysis was also performed to investigate the sensitivity of the simulated gaze estimation errors to changes in eye model parameters.

## Methods

This section describes the methodology used to simulate the real-world eye-tracking experiments performed by two comparative studies using a stochastic eye model. The section begins with a description of the comparative studies followed by the simulated environment developed to replicate the experiments performed in the comparative studies. The implementation of the stochastic eye model used to generate biometrically viable variations in eye model parameters is then discussed. Finally, the methods used to evaluate the simulated data against the findings of the comparative studies are described together with the methodology of the eye model parameter sensitivity analyses.

### Comparative Studies

The experiments conducted by the two comparative studies described in Table 1 were replicated in a simulated environment, as described in the following section. The table describes the number of participants that were included in each study, the hardware configuration of the eye-tracker used, and the types of gaze estimation algorithms investigated. The hardware configurations and gaze estimation algorithm categories are based on the categories described by Kar & Corcoran (18). The final column describes the outcomes that the eye-tracking data was used to assess.

**Table 1.** Comparative studies.

Study	Participants	Eye-tracker configuration	Gaze estimation algorithm	Evaluated
Guestrin (2006)	4	Remote	Model	Head movements
Blignaut (2014)	26	Remote	Interpolation	Algorithm comparison
				Calibration configuration

Guestrin and Eizenman (14) evaluated the performance of a model-based gaze estimation algorithm for a remote eye-tracker consisting of a single camera and two light sources during head movements. The experiment consisted of five sets of nine fixation targets that four participants were tasked with sequentially directing their gaze towards. Each set of fixations was performed at a different head position with the first fixation procedure performed in the central head position. The fixation procedure was then repeated four times with head movements of 30 mm left and right and then 40 mm forward and backwards.

The model-based gaze estimation algorithm evaluated by Guestrin and Eizenman (14) involved calibrating the parameters of a three-dimensional eye model using the extracted image features and the known positions of the hardware components of the eye-tracker. The orientation of the eye model was then taken as the direction of the user’s gaze. The radius of curvature of the cornea (*R_ac_*), the distance between the corneal centre of rotation the anatomical pupil centre (*K*), and the foveal offset (α) were calibrated by minimizing the average gaze estimation errors across the nine fixation targets in the central head position using a non-linear search algorithm. The initial parameter values suggested by Guestrin and Eizenman (14) were used for the search algorithm with *R_ac_* = 7.8 mm, *K* = 4.2 mm and α = (5, 1.5)° and η_ac_ = 1.3375. The calibrated algorithm was used to calculate the estimated gaze point for the 45 simulated fixations and the average angular gaze estimation error over the nine fixation targets in the central (H1) and 36 fixation targets in the peripheral (H2) head positions for each participant were recorded.

Blignaut (6) evaluated the performance of a large set of regression function combinations and calibration configurations on the performance of a binocular interpolation-based gaze estimation algorithm using a remote eye-tracker. Interpolation-based algorithms use extracted image features to fit some mapping function, in this case, a polynomial regression function that maps the positions of the image feature to the user’s estimated gaze position. The experiment used a remote eye-tracking configuration consisting of one camera and one infrared light source that recorded the pupil and glint features of 26 participants as they sequentially directed their gaze towards 135 fixation targets.

At each fixation, the pupil-glint vector was calculated as the vector between the pupil and glint centres and normalized by the distance between the pupils of the two eyes. The calculated pupil-glint vectors were used to calibrate twelve different regression functions using each of the six calibration configurations investigated by Blignaut (6). Calibration involved calculating the coefficients of the regression functions that minimized the gaze estimation errors over a set of calibration targets with known positions using a least squares solver. The calibration configurations used consisted of an arrangement of 5, 9, 14, 18, 23 and 135 (C5, C9, C14, C18, C23 and C135) of the 135 fixation targets. The average gaze estimation error across the 135 fixation targets was then calculated for each of the 72 calibrated regression functions as the angular error between the average estimated gaze point of both eyes and the fixation targets. The regression functions that produced the smallest average errors using each calibration configuration were identified and are referred to here as the best functions.

### Stochastic Eye Model

The first version of the aspheric stochastic model of the right eye developed by Rozema et al. (28), referred to as SyntEyes, was used in this study. The SyntEyes model was derived from the ocular biometry of 127 participants (37 male, 90 female, 123 Caucasian and 4 non-Caucasian). Various ophthalmic imaging equipment was used to capture 39 eye biometry parameters from each participant that were used to derive the 17 parameters describing the SyntEyes model. The parameters generated by the SyntEyes model are age, anterior corneal keratometry parameters (*K_ac,M,_ K_ac,J0 _*and *K_ac,J45_*), anterior and posterior corneal eccentricity (*E_ac _*and *E_pc_*), posterior corneal keratometry parameters (*K_pc,M,_ K_pc,J0 _*and *K_pc,J45_*), central corneal thickness (*CCT*), anterior chamber depth (*ACD*), anterior and posterior lens radii of curvatures (*R_al_* and *R_pl_*), lens thickness (*T*), axial length (*AL*), lens power (*D*) and low-light pupil diameter (*P_d_*).

Using Equation 1, a set of randomly generated eye models (*E*) described by *n = 17* normally distributed parameters (*b*) are generated from a multivariate Gaussian distribution using a vector (*z*) describing the mean values of the eye model parameters and a covariance matrix (*C*) describing the relationships between these parameters. The mean vector (z) and covariance matrix (*C*) provided by Rozema et al. (28) are used.

(1) $$E\left(b\right)=\;\frac{1}{{\left(2\pi \right)}^{n/2}C^{1/2}}exp\left[-\frac{1}{2}\left(b-C\right)\prime C^{-1}\left(b-M\right)\right]$$

The 17 parameters generated by the SyntEyes model were used to calculate the 14 parameters of a right eye model that includes aspheric anterior corneal surfaces, and a circular pupil with the parameters given in Table 2. Note that the corneal shape parameters are not directly generated by the SyntEyes model and were calculated as a function of the SyntEyes parameters using the equations provided by Rozema et al. (28). Given the high degree of symmetry between the eyes reported by Durr et al. (13), the same eye model parameters were used to simulate the left and right eyes of the same user. The eye model was defined relative to the optic axis with all the ocular surfaces and pupil centered on the optic axis.

**Table 2.** The parameters of the eye model generated by the SyntEyes model (28).

Parameter	Description	Calculation
*R_ac_*	Anterior corneal radius of curvature	377.2/*K_ac,M_*
*Q_ac_*	Conic constant of the anterior corneal surface	-*E_ac_^2^*
*η_ac_*	Index of refraction of the anterior corneal surface	*η(ω)_ac_*
*R_pc_*	Posterior corneal radius of curvature	1.3772-1.336/K_pc,M_
*Q_pc_*	Conic constant of the posterior corneal surface	-E_pc_^2^
*η_pc_*	Index of refraction of the posterior corneal surface	*η(ω)_pc_*
*CCT*	Central corneal thickness	*CCT*
*ACD*	Anterior chamber depth	*ACD*
*P_d_*	Pupil diameter	*P_d_*
*R_al_*	Radius of curvature of the anterior lens surface	*R_al_*
*R_pl_*	Radius of curvature of the posterior lens surface	*R_pl_*
*η_l_*	Index of refraction of the lens surfaces	*η_l_(η_ac_)*
*T*	Thickness of the lens	*T*
*AL*	Axial length of the eye model	*AL*

The refractive index of the lens and corneal surfaces were calculated as a function of the incident light wavelength ( ω) using Cauchy’s equation (2) with the chromatic dispersion coefficients used by Aguirre (1). Rozema et al. (28) caution that since the radius of the curvature of the lens surfaces (*R_al_* and *R_pl_*) and thickness of the lens (*T*) are randomly generated, the resulting power of the lens might not correspond to the lens power parameter (*D*) generated by SyntEyes. Based on the recommendation by Rozema et al. (28), the refractive index of the lens (*η_l_*) was calculated as a function of the corneal index of refraction (*η_l_*) using a derivation of the thick lens formula, given in Equation 2 with, *A = T – R_al_ + R_pl_*, to ensure that the resulting power of the lens matches the lens power (*D*) generated by the model.

(2) $$\eta_{l}=\frac{\eta_{ac}\left(T+A\right)+0.001P_{L}R_{al}R_{pl}-\sqrt{{\left(\eta_{ac}\left(T+A\right)+0.001P_{L}R_{al}R_{pl}\right)}^{2}-4T\eta_{ac}^{2}A}}{2A}$$

The SyntEyes parameters only describe the anterior segment components of the eye model. Posterior segment parameters were required to perform fixations of the eye model. The posterior chamber parameters of the eye model proposed by Aguirre (1) were used. This includes an ellipsoidal retina surface, an offset angle of the fovea from the optic axis (*α*), separate centres of rotation for azimuth (*E_r,z_*) and elevation (*E_r,y_*) eye rotations. Aguirre (1) scaled these parameters according to the spherical refraction (SR) of the eye model and the laterality of the eye being simulated. Since the SyntEyes model does not generate a value of spherical refraction, the spherical refraction (*SR*) of the eye model was calculated as *SR(AL) = (23.58 - AL)/0.299* according to the relationship proposed by Atchison (3).

### Simulation Procedure

The MATLAB-based gkaModelEye framework (1) was used to develop the simulation environment used in this investigation. The framework contains computational models of the various components present in an eye-tracking experiment including an eye, camera, light sources, and screen. The framework uses ray tracing operations to simulate the image features observed by the camera of an eye-tracker such as the glint and apparent pupil for various orientations of the eye model. This framework was preferred over other options such as the et_simul (8) and UnityEyes (32) frameworks as it readily facilitates the inclusion of a high-complexity eye model and provides the functionality to configure the simulation environment to replicate the experiments performed by the comparative studies.

**Figure 1.**
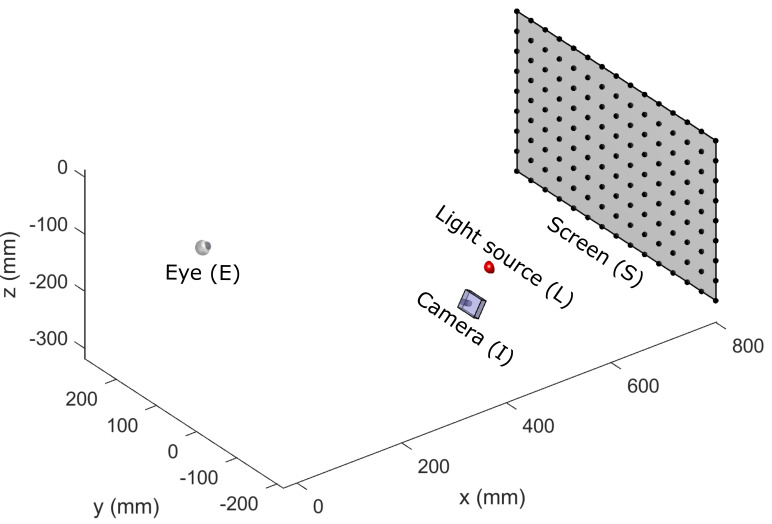
An illustration of the simulation environment that was configured to replicate the experiment performed by Blignaut (6). The black dots on the screen represent the fixation targets that participants were asked to focus their gaze on.

The configuration of the simulation environment developed was defined by the nine parameters described in Table 3. The configurations of the parameters that were used to replicate the experiments performed by the comparative studies investigated in this chapter are also given in the table. Figure 1 illustrates the graphical output of the simulation environment configured to replicate the experimental configuration used by Blignaut (6). The origin of the simulation environment was placed on the intersection of the optic axis with the anterior corneal surface of the eye in an unrotated orientation. The x-axis was directed along the optic axis of the eye towards the screen, the y-axis was directed nasally for the right eye and temporally for the left eye and the z-axis was directed superiorly.

**Table 3.** The parameters of the simulation environment and the configurations of the environment used to replicate each comparative study.

Parameter	Description	Guestrin et al. (2006)	Blignaut (2014)
I_t_ (mm)	Position of the camera’s nodal point	(315, IPD*/2, -150.5)	(520, IPD*/2, -300)
I_r_ (°)	Rotation of the camera around its nodal point	(0, 13.8, 0)	(0, 30, 0)
I_s_ (mm)	Dimensions of the camera’s image sensor	(4.8, 3.6)	(4.48, 3.36)
I_fl_ (mm)	Focal length of the camera	35	10
L_t,1_ (mm)	Position of the first point light source	(615, IPD*/2 + 188.5.5, 0)	(560, IPD*/2, -250)
L_t,2_ (mm)	Position of the second point light source	(615, IPD*/2 - 188.5.5, 0)	
L_w_ (nm)	Illumination wavelength of the light source	850	850
S_t_ (mm)	Position of the centre of the screen	(650, IPD*/2, 0)	(800, IPD*/2, -131.5)
S_s_ (mm)	Dimensions of the screen	(377, 301)	(495, 280)
S_f_ (targets)	Configuration of the fixation targets	(3, 3)	(15, 9)

*IPD – Interpupillary distance

The fixation procedure performed in the eye-tracking experiments was simulated by sequentially fixating the eye model on a series of fixation targets and then using ray tracing operations to simulate the position of the glint and apparent pupil centres on the image sensor of the camera model. Each fixation was performed by rotating the eye model in accordance with Listing’s law around its centres of rotation (*E_r,z_* and *E_r,y_*) so that its line of sight aligns with the fixation target (1). This is a non-trivial operation as the position of the entrance pupil’s centre to which the line-of-sight is defined changes non-linearly as the eye rotates (24). The gkaModelEye framework (1) uses a non-linear search algorithm to find the orientation of the eye that results in a line-of-sight that intersects the fixation target as well as the entrance pupil’s centre and the fovea of the eye model.

During each fixation, the position of the apparent pupil boundary was simulated by projecting 30 points uniformly arranged around the anatomical pupil boundary through the cornea so that the rays intersect the nodal point of the camera and its image sensor. The apparent pupil’s centre was then calculated as the centre of an ellipse fitted to the projected apparent pupil boundary points using a least squares solver. The glint centres were simulated as the intersection of rays with the image sensor that project from each point light source, reflects off the anterior corneal surface and then intersects the nodal point of the camera. All ray traces performed in this study were confirmed to intersect the nodal point of the camera within $10^{-4}$ mm.

The simulation environment only included one eye model at a time. Binocular eye-tracking data of a single user was generated by performing the fixation procedure twice with the same eye model in a different position. The right eye of a user was simulated by translating the screen, camera, and light source components half of the interpupillary distance (*IPD*) along the positive y-axis. Conversely, a left eye was simulated by translating the same components by half of the interpupillary distance along the negative y-axis. A fixed interpupillary distance of 63 mm was used in all the simulations based on the interpupillary distance of the average human reported by Dodgson (11). The interpupillary distance was kept constant so that variations in eye model parameter were the only influence on gaze estimation outcomes.

**Figure 2.**
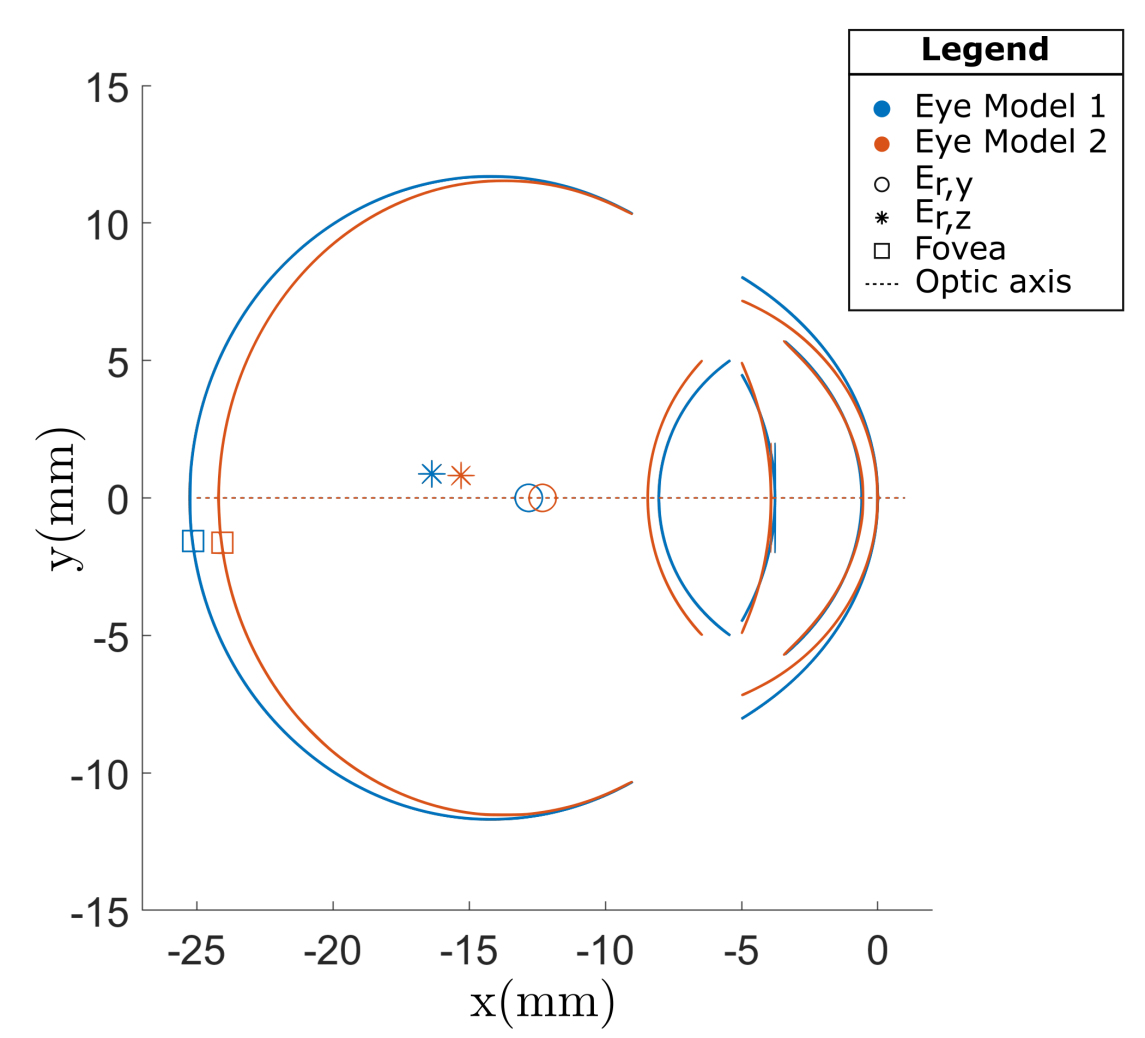
A comparison of two eye models that were generated using the SyntEyes model. The orange eye model has a smaller axial length, slightly larger anterior chamber depth and a more aspheric cornea than the eye model shown in blue.

The stochastic eye model was used to generate 100 unique eye models. The fixation procedures performed in the two comparative studies were then simulated using each of the 100 eye models. An example of two superimposed eye models generated with the SyntEyes model is illustrated in Figure 2. The eye model shown in orange has a shorter axial length, slightly larger anterior chamber depth and more aspheric cornea than the eye model shown in blue. The average gaze estimation errors at the central (H1) and peripheral (H2) head positions was then calculated for the comparative study by Guestrin and Eizenman (14) and the 12 regression functions calibrated using each of the six calibration configurations for the comparative study by Blignaut (6).

### Analyses

The distribution of the eye model parameters generated by the SyntEyes model (28) were inspected to ensure that the model was correctly implemented in the simulation environment, The simulated pupil and glint centres were then plotted to inspect the influence of eye model parameter variations on the simulated image features.

The simulated gaze estimation errors were compared to the real-world eye-tracking errors reported by Guestrin and Eizenman (14), and Blignaut (6). Two-tailed student t-tests were performed to compare the mean simulated and real-world gaze estimation errors and a two-tailed F-test was performed to compare the variance in errors simulated to the errors reported in the real world reported by Guestrin and Eizenman (14. A single sample student t-test was performed to compare the simulated and real-world gaze estimation errors generated by the experiment performed by Blignaut (6).

The simulated data was also investigated to identify any significant relationships between the simulated gaze estimation errors and the 11 eye model parameters given in Table 2 excluding the indexes of refractions (*η*). A multivariate regression analysis was performed using Matlab (v2019b, Mathworks, USA). The multivariate regression function is given in Equation 2 with the parameter matrix (*B*) containing the *h = 11* parameters of the *n = 100* eye models that were used to generate the simulated data. The parameter matrix (*B*) was converted to z-scores by subtracting the mean value of each parameter column from each element in the column and then dividing it by the standard deviation of the column. The error matrix (*W*) consisted of *t = 8* columns representing the gaze estimation errors categories simulated for the two comparative studies. The error categories were chosen to correspond to the results reported by the comparative studies with *l = 1, 2* representing the errors generated at H1 and H2 by simulating the experiment performed by Guestrin and Eizenman (14). The remaining columns, *l = 3 … 8* represented the best functions’ errors simulated using each of the six calibration configurations evaluated by Blignaut (6).

(2) $$B_{i,q}a_{q,l}=W_{i,l};\;\;i\in [0,n],\;q\in [0,h],\;l\in [0,t]$$

By solving for the coefficients *a_q,l_*, the value of each coefficient describes the sensitivity (*m*) of the simulated gaze estimation errors in the corresponding error category (*W_l_*) to changes in the eye model parameters (*q*) in units of standard deviations. The *ρ* parameter of each regression coefficient in the resulting multivariate regression model was inspected for eye model parameters that have a significant influence (ρ < .05) on the simulated gaze estimation errors of each error category (*l*). The coefficients *a_q,l _*were again solved using only the significant eye model parameters.

A partial regression plot was generated for each error category (*l*) for the constant term against the residuals of the regression model that includes the terms for all the significant eye model parameters. Partial regression plots are commonly used in multivariate regression analyses to analyze the influence that the addition of an independent variable has on the residuals of a regression model that includes terms for all the other independent variables being considered (35). Therefore, a partial regression plot for the constant term of a regression model visualizes the performance of the entire model.

The R^2^ error of each regression model was calculated to evaluate the variance in simulated gaze estimations that were captured by the significant eye-model parameters for each error category (12). An R^2^ value of one indicates that the significant eye model parameters completely described the variance in simulated gaze estimation errors with no influence from the other eye model parameters. Deviations from one define the magnitude of contributions from non-significant eye model parameters or a non-linear relationship between errors and eye model parameters.

## Results

In this section, the simulated outcomes are evaluated against the experimental findings of the comparative studies and the relationships between eye model parameters and the simulated eye-tracking outcomes are described.

### Eye Model Parameters

The distribution of the eye model parameters of the 100 eye models generated by the SyntEyes model (28) are given in Table 4. The mean values of the parameter distributions correspond closely to the mean values of the SyntEyes model (28). This demonstrates that the eye model was correctly implemented in the simulation.

**Table 4.** The distribution of the eye model parameters of the 100 eye models generated by the SyntEyes model (28).

Parameter	Outcome
*R_ac_*	7.79 ± 0.24 mm
*Q_ac_*	-0.21 ± 0.16
*η_ac_*	1.34
*R_pc_*	6.52 ± 0.24 mm
*Q_pc_*	-0.11 ± 0.17
*η_pc_*	1.34
*CCT*	0.545 ± 0.037 mm
*ACD*	3.41 ± 0.37 mm
*P_d_*	6.44 ± 1.20 mm
*R_al_*	10.47 ± 1.32 mm
*R_pl_*	6.96 ± 0.89 mm
*η_l_*	1.44 ± 0.01
*T*	4.00 ± 0.51 mm
*AL*	23.74 ± 1.07 mm

### Simulated Image Features

**Figure 3.**
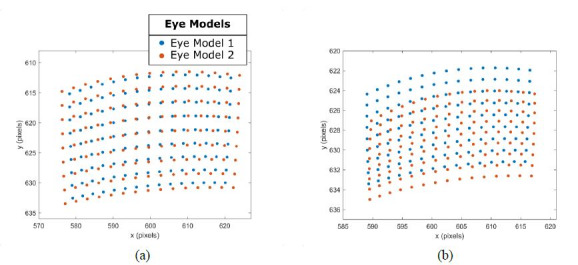
The simulated (a) pupil and (b) glint features generated for two eye models.

The influence that variations of eye model parameters have on the simulated image features are illustrated in Figure 3. The blue and orange image features were simulated using the correspondingly colored eye models in Figure 2 with the eye models sequentially fixated on 135 fixation targets in the simulation environment shown in Figure 1.

A clear difference between the simulated pupil and glint centres can be observed. The pupil centre features of Eye Model 2 have a larger distribution than the features of eye model 1. The aspect ratio of the glint features for Eye Model 2 is flatter and translated in the positive y-axis relative to the glint features simulated using Eye Model 1.

### Guestrin and Eizenman (2006)

**Figure 4.**
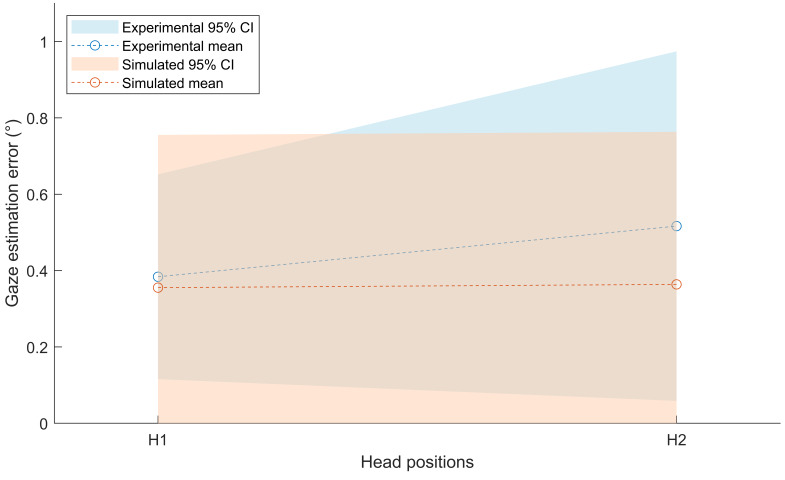
A comparison of the real-world outcomes of the study performed by Guestrin and Eizenman (14) and the simulated outcomes achieved in this study.

The mean and 95% confidence intervals (CI) of the simulated outcomes generated in this study for the comparative study by Guestrin and Eizenman (14) are compared to the real-world outcomes at H1 and H2 in Figure 4. The simulated outcomes at both head positions were found to be strongly right-skewed (H1: skewness = 1.86, H2: skewness = 1.84). This indicates that the simulated gaze estimation errors were more strongly concentrated towards smaller errors than the normal distribution illustrated in Figure 4 suggests. However, the simulated errors are reported as a normative distribution to facilitate the comparison to experimental results.

The simulated mean errors at both head positions (H1: M = 0.36, SD = 20; H2: M = 0.36, SD = 20) were not significantly different from the real-world outcomes (H1: *t*(4.47) = 0.21, *ρ* = .84; H2: *t*(3.47) = 1.79, *ρ* = .16). The variances in eye-tracking errors between the simulated and real-world outcomes were also not significantly different (H1: F(99,3) = 5.53, *ρ* = .18; H2: F(99,3) = 1.89, *ρ* = .67). However, the simulated data was not able to predict the increase in the mean or variance of errors during head movements observed in the real-world data.

**Figure 5.**
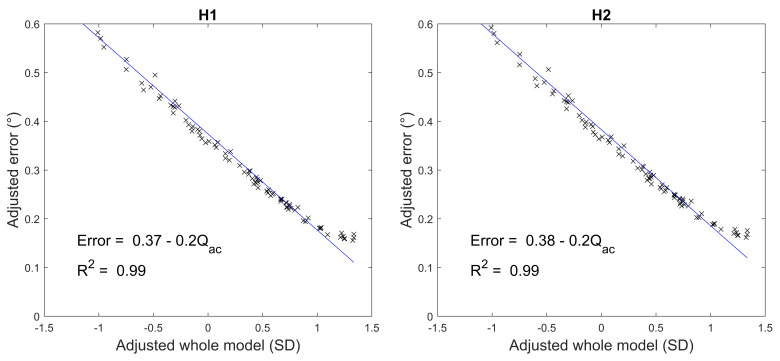
The influence of the anterior corneal asphericity on the gaze estimation errors generated by the simulation of the real-world experiment performed by Guestrin and Eizenman (14).

Only the anterior corneal asphericity (Q_ac_) demonstrated a statistically significant influence (*ρ* < .05) on the simulated gaze estimation errors at each head position, as illustrated in Figure 5. A significant sensitivity of 0.2° error per standard deviation (SD) change in anterior corneal asphericity was found at both head positions. Figure 5 further shows that the variations in errors are well described by variations in anterior corneal asphericity with R^2^ = .99 at both head position categories.

### Blignaut (2014)

**Figure 6.**
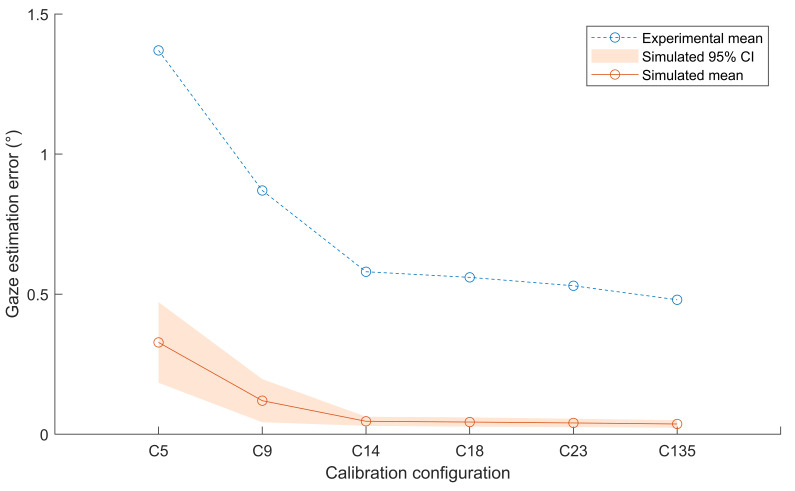
A comparison of the real-world outcomes of the study performed by Blignaut (6) and the simulated outcomes achieved in this study.

The distribution of the simulated and experiential best functions errors are illustrated in Figure 6. The simulated best function errors were significantly different from the experimental outcomes using each calibration configuration (*t*(25) > 5.34, *ρ* < .05 for all tests).

The simulated data predicted the negligible relative decrease in errors achieved by increasing the number of calibration targets beyond 14 as observed in the experimental data. The real-world decrease in the best functions’ errors was 0.28° and the simulated decrease was 0.20° for an increase from five to nine calibration targets. All subsequent increases in calibration targets resulted in a change in errors of under 0.1° in both the real-world and simulated errors.

The variances of the simulated and real-world outcomes could not be compared as the variance of the real-world outcome was not reported by Blignaut (6). However, an interesting observation can be made from Figure 6. The figure shows that the variance in simulated errors decreased with each addition of calibration targets up to C14. This indicates that as the number of calibration targets was increased, the best functions were better able to account for the interpersonal variations in image feature distributions. After C14, the best functions could almost completely compensate for interpersonal variations in eye model parameters.

**Figure 7.**
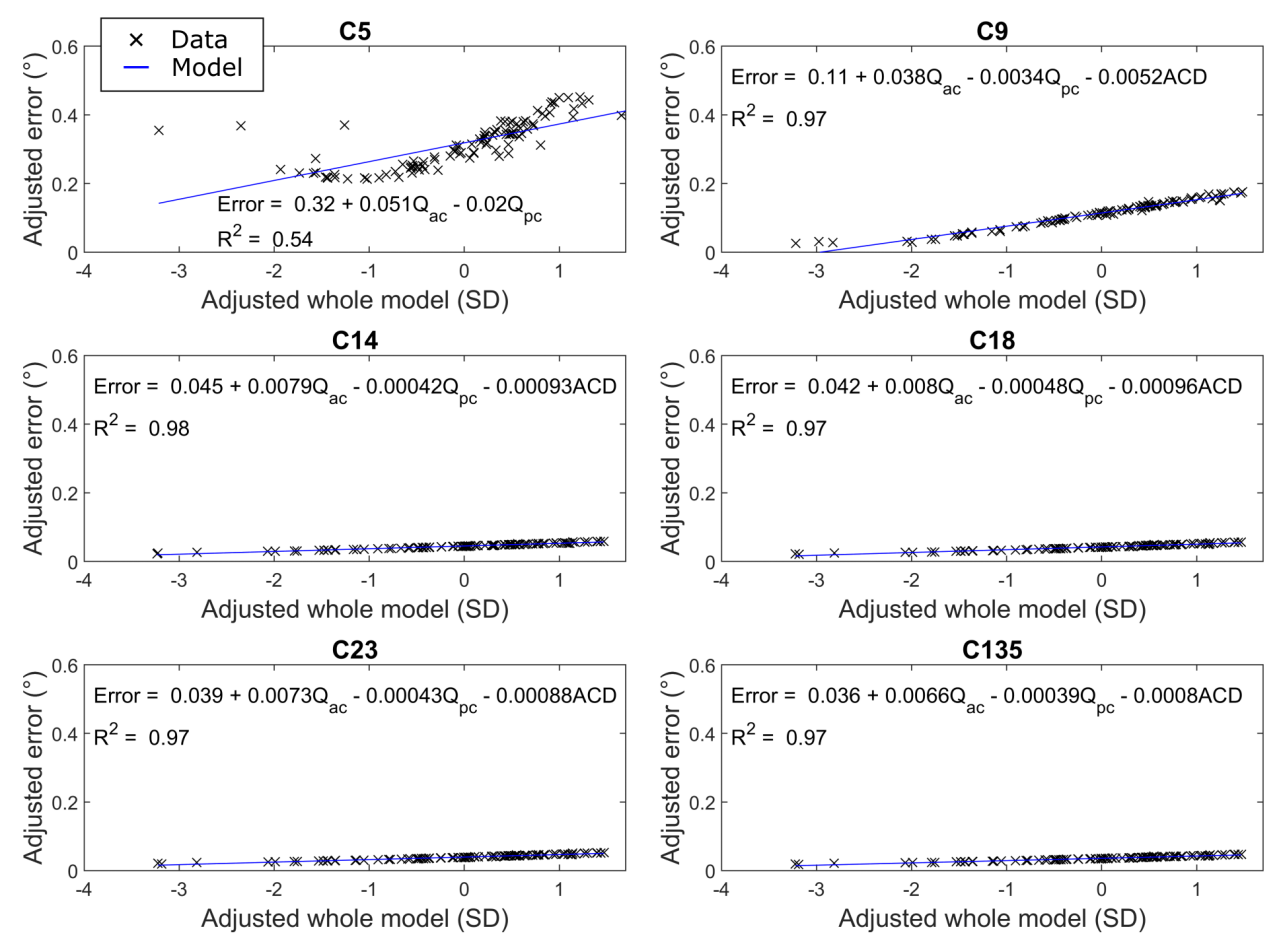
The influence of eye model parameters on the simulated outcomes generated by the real-world experiment performed by Blignaut (6).

The relationship between eye model parameters and simulated gaze estimation errors given in Figure 7 further clarifies the observed error variances over the number of calibration targets. The strongest influence on errors was the anterior corneal asphericity (Q_ac_) with a smaller but not insignificant contribution by the anterior chamber depth (*ACD*) and posterior corneal asphericity (Q_pc_) at each calibration configuration.

## Discussion

A discussion of the findings of this study is provided in this section. The discussion starts with an overview of the implications of the findings of the study. The limitations of this investigation are then described followed by recommendations for future work.

### Implications of Findings

The simulated mean and the variance in errors were statistically similar to the experiential outcomes reported for the model-based gaze estimation algorithm evaluated by Guestrin (14). This indicates that the stochastic eye model provided a good prediction of how the real-world eye-tracking outcomes produced by model-based gaze estimation algorithms are influenced by interpersonal variations in the shape of the eye. The simulated mean best functions errors were significantly different than the experimental results reported by Blignaut (6). However, the simulated and real-world interpolation-based gaze estimation errors followed a similar trend when an increasing number of calibration targets were used.

The most influential eye model parameter on the simulated gaze estimation errors in both comparative studies was the anterior corneal asphericity (Q_ac_). The anterior chamber depth (*ACD*) and posterior corneal asphericity (Q_pc_) also had a smaller but statistically significant influence on simulated interpolation-based gaze estimation errors. This indicates that studies aiming to simulate the interpersonal variations in the eye-tracking performance of an interpolation or model-based gaze estimation method should simulate variations in the asphericity of the cornea.

This finding is alarming considering that variations in anterior corneal asphericity are rarely considered in simulated eye-tracking data (32; 19; 27). Other eye model parameters that are more frequently varied in the existing literature have been the anterior corneal radius of curvature and the foveal offset (21; 10; 25), the first of which was shown in this chapter to have a negligible influence on simulated eye-tracking outcomes. The foveal offset was not explicitly investigated in this study. However, the foveal offset was scaled relative to the axial length (*AL*) of the eye and a significant influence would’ve been captured by this parameter.

### Limitations

The distribution of eye model parameters generated by the SyntEyes model is dependent on the distribution of the eye biometry used to derive the model. The population used to generate the SyntEyes model was slightly skewed towards women and highly skewed towards Caucasians. This may introduce some bias in the simulated eye-tracking outcomes and the findings of this study towards these populations.

The first version of the SyntEyes model is a paraxial model intended to replicate the central visual field of the eye. The maximum viewing angle of the eye model was just under 20° in the simulation of both comparative studies. Studies such as Polans et al. (26) have shown that paraxial eye models fail to accurately replicate the visual performance of the eye at the peripheral viewing angles used in this study as they extrapolate centrally determined eye parameters to the periphery of the ocular surfaces and do not include realistic ocular surface decentrations and tilts. The risk of extrapolating central eye parameters are illustrated in Eye Model 2 in Figure 2 where the cornea becomes thinner towards its periphery which is not physiologically accurate (4).

Rozema et al. (29) developed the second version of the SyntEyes stochastic eye model. This model was derived from a larger and more diverse population and includes corneal surfaces described by an 8th-order Zernike polynomial. Unfortunately, this patient-specific eye model could not be used in this study as the Zernike coefficients are only provided for the central 6.5 mm of the corneal surfaces. This was not sufficient for simulating eye-tracking outcomes at large viewing angles as the pupil and glint rays may not interest the corneal surfaces.

Interpersonal variations in the pupil centre position and translation of the apparent pupil’s centre during changes in the size of the pupil have been shown to adversely influence the performance of eye-trackers (9). Pupil centre shifts were not included in this investigation as it is unclear the extent to which shifts in the apparent pupil’s centre are caused by the influence of the corneal optics as the anatomical pupil’s diameter changes or asymmetrical dilations and constriction of the anatomical pupil.

### Future Work

The work presented in this study demonstrated a method to readily include biometrically plausible eye model parameter variations in simulated data using a stochastic eye model. This method has significant potential to increase the distributions of realistic interpersonal variations contained in synthetic image datasets. Supplementing the training of appearance-based gaze estimation algorithms with a dataset that contains interpersonal variations in eye biometry could significantly improve the outcomes achieved by these algorithms.

The realism of simulated eye-tracking outcomes could be further improved by using a higher complexity stochastic eye model that includes patient-specific corneal surface parameters as well as ocular decentrations and tilts. Once a higher complexity stochastic eye model becomes available, the multivariate regression analysis method performed in this study can be repeated to gain further insight into how interpersonal variations in the anatomy of the eye influence eye-tracking outcomes. Improving the complexity of the eye model could also improve the similarity of the simulated outcomes to the experimental outcomes generated by interpolation-based gaze estimation algorithms.

### Ethics and Conflict of Interest

The author(s) declare(s) that the contents of the article are in agreement with the ethics described in http://biblio.unibe.ch/portale/elibrary/BOP/jemr/ethics.html and that there is no conflict of interest regarding the publication of this paper.

### Acknowledgements

“Research reported in this publication was supported by the South African Medical Research Council under a Self-Initiated Research Grant. The views and opinions expressed are those of the authors and do not necessarily represent the official views of the SA MRC. This work is also based on the research supported in part by the National Research Foundation of South Africa (Grant Numbers: 113337)
